# Smooth muscle titin forms *in vitro* amyloid aggregates

**DOI:** 10.1042/BSR20160066

**Published:** 2016-05-20

**Authors:** Alexandr G. Bobylev, Oxana V. Galzitskaya, Roman S. Fadeev, Liya G. Bobyleva, Darya A. Yurshenas, Nikolay V. Molochkov, Nikita V. Dovidchenko, Olga M. Selivanova, Nikita V. Penkov, Zoya A. Podlubnaya, Ivan M. Vikhlyantsev

**Affiliations:** * Laboratory of Structure and Functions of Muscle Proteins, Institute of Theoretical and Experimental Biophysics, Russian Academy of Sciences, 142290 Pushchino, Moscow Region, Russian Federation; † Group of Bioinformatics, Institute of Protein Research, Russian Academy of Sciences, 142290 Pushchino, Moscow Region, Russian Federation; ‡ Laboratory of Cryobiology and Biophysics of Water, Institute of Cell Biophysics, Russian Academy of Sciences, 142290 Pushchino, Moscow Region, Russian Federation

**Keywords:** amyloid, amyloid aggregates, cytotoxicity, smooth muscle titin

## Abstract

Amyloids are insoluble fibrous protein aggregates, and their accumulation is associated with amyloidosis and many neurodegenerative diseases, including Alzheimer's disease. In the present study, we report that smooth muscle titin (SMT; 500 kDa) from chicken gizzard forms amyloid aggregates *in vitro*. This conclusion is supported by EM data, fluorescence analysis using thioflavin T (ThT), Congo red (CR) spectroscopy and X-ray diffraction. Our dynamic light scattering (DLS) data show that titin forms *in vitro* amyloid aggregates with a hydrodynamic radius (Rh) of approximately 700–4500 nm. The initial titin aggregates with Rh approximately 700 nm were observed beyond first 20 min its aggregation that shows a high rate of amyloid formation by this protein. We also showed using confocal microscopy the cytotoxic effect of SMT amyloid aggregates on smooth muscle cells from bovine aorta. This effect involves the disorganization of the actin cytoskeleton and result is cell damage. Cumulatively, our results indicate that titin may be involved in generation of amyloidosis in smooth muscles.

## INTRODUCTION

Amyloids are insoluble fibrous protein aggregates sharing specific structural features: high content of β-sheet structure, ability to bind to Congo red (CR) and thioflavin T (ThT), birefringence in polarized light, insolubility in most solvents and resistance to proteases [1–3]. Amyloids can be divided into two classes: fibrils with highly ordered structures and amorphous aggregates. More than 50 proteins had been described to form amyloids which are involved in pathogenesis of amyloidosis in different organs and tissues, for example tau-protein, hantingtin, amylin, amyloid-β (Aβ) peptide, α-synuclein, insulin, lysozyme, myoglobin, transthyretin. [3,4]. However, there is a group of proteins whose amyloid properties were demonstrated *in vitro*, that are not associated with known diseases [3,4]. Amyloid was found in cross-striated muscles, for example formed from such proteins as immunoglobulin (Ig) light chain, Ig heavy chains, transthyretin, serum amyloid A, apolipoprotein AIV, fibrinogen α chain and atrial natriuretic factor, which contribute to the development of ‘amyloid cardiomyopathy’ or ‘cardiac amyloidosis’ [5]. Amyloid depositions containing the Aβ peptide were detected upon generation of inclusion-body myositis in skeletal muscles. [3]. It was also found that myosin subfragment-1 from rabbit skeletal muscles can form *in vitro* spherical oligomers with amyloid-like dye-binding properties [6]. Amyloid deposits were also found in blood vessels [7,8]; in particular, it was demonstrated that aggregates of serum amyloid A (SAA) (an amyloid-related protein from human serum) and its fragments can accumulate in the intima and medial arterioles and beneath the venular endothelium [7]. Medin amyloids (50-aa-long peptides of lactadherin) were detected at aorta amyloidosis [8].

Titin (connectin) is a giant elastic protein of striated [9–12] and smooth [13] muscles of vertebrates, discovered at the end of the last century [14,15]. Titin is the third most abundant protein (after actin and myosin) in sarcomeres of cardiac and skeletal muscles of vertebrates. Its molecules of approximately 1 μm in length and 3–4 nm in diameter [16] overlap the half of the sarcomere from the M-line to the Z-line, forming a third filamentous system in myofibrils [17]. The molecular mass of titin isoforms (N2A, N2BA and N2B) and their isovariants are 3000–3700 kDa [12,18,19]. Studies conducted over the past 20 years have shown that titin is one of the key components of the sarcomere of vertebrate striated muscles, and it plays an important role in the assembly of thick filaments, formation of highly ordered sarcomere structure, regulation of actin–myosin interaction and intracellular signalling processes (see reviews [12,20]). Nevertheless, not all of the functional properties of titin and structural peculiarities of its different isoforms have been thoroughly studied. This is due not only to the relative ‘youth’ of titin as an object of research, but also to methodical difficulties of studying a protein with such high molecular mass. The ability of titin to be easily degraded during preparative procedures also makes it very difficult to study its structure–functional properties.

In 2002, smooth muscle titin (SMT; initially called smitin) was found in the smooth muscle extract of chicken gizzard [21]. Further studies showed that titin from smooth and cross-striated muscles is a product of the same gene, alternative splicing of which leads to the formation of isoforms of 700–2000 kDa in smooth muscle [13]. Western blotting demonstrated titin of approximately 500 kDa in human aorta, which the authors [13] suggested is either a titin fragment (‘truncated’ titin) or an isoform.

As is well known, titin from cross-striated and smooth muscles consists of immunoglobulin (Ig)-like and fibronectin III (FnIII)-like domains with a β-sheet structure. It was demonstrated that approximately 10% of Ig and FnIII domains have more than 40% sequence identity [22]. It was also shown by dynamic light scattering (DLS) that the aggregation rate of TI I32 and TI I27 domains with sequence identities of 42% is higher than that of domains with a lower sequence identity [22]. In a recent study conducted by microfluidic-mixing single-molecule kinetics, ensemble experiments and molecular simulations to investigate how misfolding between the Ig-like domains of titin is prevented, it has been shown, that during refolding of tandem repeats, independent of sequence identity, more than half of all molecules transiently form a wide range of misfolded conformations [23]. Simulations suggest that a large fraction of these misfolds resemble an intramolecular amyloid-like state reported in computational studies [23]. In our own studies, we showed the ability of myosin binding protein-C containing Ig-like and Fn-like domains to form aggregates with amyloid-like properties *in vitro* [24]. Based on the data obtained, we proposed that SMT can form amyloid aggregates *in vitro*. In the present study, we present data from the 500 kDa SMT isoform, supporting the above proposal, and also the results of examination of the cytotoxicity of SMT aggregates in order to clarify their potential negative effect, similar to Aβ(1–42)-peptide oligomers.

## MATERIALS AND METHODS

### Purification of chicken gizzard smooth muscle titin

SMT was purified from fresh chicken gizzard by the method described in [21] with modification. Fresh chicken gizzard smooth muscles were diced and homogenized for 10 s with a Waring blender in buffer A (50 mM KCl, 2 mM MgCl_2_, 1 mM EDTA, 1 mM EGTA, 0.5 mM DTT, 0.2 mM PMSF and 0.1% cocktail protease inhibitors, 10 mM imidazole, pH 7.0). The myofibrils were pelleted by centrifugation (5000 ***g*** for 10 min, 4°C), washed three times with buffer A and resuspended in extraction buffer (0.6 M KCl, 4 mM ATP, 2 mM MgCl_2_, 1 mM EDTA, 1 mM EGTA, 0.5 mM DTT, 0.2 mM PMSF and 0.1% cocktail protease inhibitors, 10 mM imidazole, pH 7.0) for 60 min (final ionic strength approximately 0.4). The extract was clarified for 30 min at 15000 ***g*** and the supernatant diluted 2-fold with distilled (4°C) water containing 0.1 mM DTT and 0.1 mM NaN_3_ to precipitate actomyosin (final ionic strength approximately 0.2). After 1 h, the supernatant was clarified for 60 min at 20000 ***g***. To pellet SMT, the supernatant was further diluted 4-fold (final ionic strength approximately 0.05) with ice-cold distilled water containing 0.1 mM DTT and 0.1 mM NaN_3_. After 40–60 min, the pellet containing mostly SMT was collected by centrifugation for 30 min at 15000 ***g***. The pellet was dissolved in a minimal volume of buffer containing 0.6 M KCl, 30 mM KH_2_PO_4_, 1 mM DTT, 0.1 M NaN_3_, pH 7.0 and clarified for 60 min at 20000 ***g***. SMT was further purified by gel-filtration on a Sepharose-CL2B column equilibrated in the same buffer.

### Gel electrophoresis and Western blotting

SDS/PAGE was carried out as described in [25] with our modification. The separating gel with 7% polyacrylamide was used [25]. The stacking gel contained 4% polyacrylamide and was prepared as described in [26]. Western blotting was performed as described in [27]. 9D10 anti-vertebrate striated muscle mouse monoclonal antibody was used to detect titin. Mouse IgG antibodies (Sigma–Aldrich) were used as secondary antibodies conjugated with horseradish peroxidase.

### EM and conditions for amyloid formation

SMT aggregates were formed during 24 h at 4°C against a solution containing 0.15 M glycine–KOH, pH 7.0–7.5. A drop of suspension at the 0.1 mg/ml was applied to a carbon-coated collodion films on copper grids and negatively stained with 2% aqueous uranyl acetate. Samples were examined on JEM-100B and LIBRA 120 ‘Carl Zeiss’ electron microscopes.

### CD method

SMT (concentration 0.4 mg/ml in solution containing 0.6 M KCl, 30 mM KH_2_PO_4_, 1 mM DTT, 0.1 M NaN_3_, pH 7.0) was dialysed for 24 h using dialysis tubing cellulose membrane (Sigma–Aldrich) against the buffer containing 0.15 M glycine–KOH, pH 7.0–7.5. CD spectra prior to and after SMT aggregation were recorded in a Jasco J-815 spectrometer (JASCO) using 0.1 cm optical path-quartz cells and wavelengths 250–190 nm. Secondary structure was calculated using the CONTINLL module of the CDPro program [28].

### Fluorescence analysis with thioflavin T

Amyloid was estimated by ThT fluorescence intensity in samples containing 0.15 M glycine–KOH, pH 7.0 and 0.05 M glycine–KOH, pH 7.0, 5 μM ThT using 50 μg/ml SMT. Fluorescence was measured at *λ*_ex_=440 nm and *λ*_em_=488 nm using a Cary Eclipse spectrophotometer (Varian).

### Congo red

To verify the nature of SMT aggregates, the amyloid-specific stain CR was also used. SMT aggregate suspension (250 μl, 0.2 mg/ml) was added to 250 μl 0.15 M glycine–KOH, pH 7.0–7.5 with CR (0.1 mg/ml). The final ratio of the protein: CR in the sample was 2:1 (w/w). Absorption spectra of CR in the absence and presence of the protein were recorded at 450–650 nm using a CARY 100 spectrophotometer (Varian).

### X-ray diffraction

SMT for X-ray diffraction analysis was prepared after 24 h incubation at 4°C in solution containing 0.15 M glycine–KOH, pH 7.0–7.5. Protein at 0.044 mg/ml (*C*_0_) and volume (*V*_0_) of 1.4 ml was concentrated 100-fold to 14 μl and 4.4 mg/ml using an Eppendorf 5301 vacuum concentrator. The pellet at the tube bottom (4 μl) was dissolved in 10 μl of the same buffer. Droplets of this preparation were placed between the ends of wax-coated glass capillaries (approximately 1 mm in diameter) separated approximately by 1.5 mm. Fiber diffraction images were collected using a Microstar X-ray generator with HELIOX optics, equipped with a Platinum135 CCD detector (X8 Proteum system, Bruker AXS) at the Institute of Protein Research, RAS, Pushchino. Cu Kα radiation, *λ*=1.54 Å (1 Å=0.1 nm), was used. The samples were positioned at the right angle to the X-ray beam using a four-axis kappa goniometer.

### Dynamic light scattering experiments

SMT samples for DLS were prepared as follows: Purified SMT at 0.2 mg/ml in column buffer (0.6 M KCl, 30 mM KH_2_PO_4_, 1 mM DTT, 0.1 M NaN_3_, pH 7.0) was dialysed (size 25×16 mm, Sigma–Aldrich cellulose membrane tubing) at 4°C against 0.15 M glycine–KOH, pH 7.0–7.5. Samples for DLS were collected after: 20, 40, 60, 120 and 180 min. The last sample was left in the instrument for continuous measurements over 21 h. In this experiment, the SMT preparation in the column buffer was the initial point (0 min). As viscosity of the SMT preparations varied due to different buffer composition, and the calculations of dimensions based on DLS depend on viscosity, solution viscosities were measured in order to determine more correctly the hydrodynamic radius (Rh) of the particles. For this, we determined the product of the dynamic viscosity from the density of the titin solution containing in column buffer (0.6 M KCl, 30 mM KH_2_PO_4_, 1 mM DTT, 0.1 M NaN_3_, pH 7.0) which was 1.39 sP × g/cm^3^. After measuring the solution density (1.025 g/cm^3^), we calculated the dynamic viscosity value (1.36 cP). This value was taken into account in measurements of particle dimensions in SMT samples collected 20, 40, 60 and 120 min after the dialysis. The product of the dynamic viscosity by the density of solution (1 g/cm^3^) containing SMT in 0.15 M glycine–KOH buffer (pH 7.0–7.5) was 0.92 cP × g/cm^3^. Correspondingly, the dynamic viscosity value was 0.92 cP. This value was taken into consideration when measuring the particle dimensions in SMT samples collected 180 min after the dialysis. DLS was performed in a Zetasizer Nano ZS (Malvern Instruments) with 4 mW He–Ne laser (632.8 nm) and sample cuvette temperature control (10°C). After thermal equilibration (typically 5 min), autocorrelation functions were collected every 15 runs, using run acquisition times of 15 s. Autocorrelation functions were converted into particle-size distributions, using the ‘General purpose’ algorithm provided with the Zetasizer. Particle-size distributions obtained from alternative inversion algorithms yielded comparable results. Dynamic viscosity of solutions was determined using a Sine-wave Vibro Viscometer SV-10 (A&D Company). Density was determined by weighing 1 ml of the solution.

### Cytotoxicity assay

To study cytotoxicity of amyloid SMT aggregates, the protein was lyophilized using a FreeZone 1l lyophilizator (Labconco). 1% trehalose was used as a stabilizing agent. SMT quality and any degradation after lyophilization were monitored using 7% SDS/PAGE [25]. Cytotoxicity was evaluated with smooth muscle cells isolated from bovine aorta as described in [29] using crystalline violet assay [30]. The cells were seeded into 96-well cell culture plates (Greiner) at density of 3000 cells per well. Cell were cultured in DMEM/F12 Nutrient Mixture (Sigma–Aldrich) with 10% FBS (Gibco), 40 μg/ml gentamicin sulfate (Sigma–Aldrich) to the confluent state, at 37°C in an atmosphere containing 5% CO_2_. After the confluent formation, cells were incubated in the DMEM/F12 without serum for 2 h. SMT was added in the molecular or aggregated form. F-actin was used as a control. Cytotoxicity was estimated from the difference between absorbance in the experiment and background to the difference between the control absorbance and background in 72 h incubation. Absorbance was proportional to the number of live cells. The measurements were performed using an Infinite F200 plate reader (Tecan).

### Confocal microscopy

Bovine aorta smooth muscle cells were plated on coverslips in six-well culture dishes. After 24 h DMEM/F12 with 10% FBS was substituted by a serum-free medium and 2 h later aggregated SMT was added. F-actin was used as a control. After 48 h incubation at 37°C in 5% CO_2_, the cells were washed with cold PBS three times, fixed with 4% paraformaldehyde (PFA) for 2 h at room temperature and then permeabilized with 0.1% saponin (Sigma–Aldrich). The staining of the smooth muscle actin cytoskeleton was performed in medium containing PBS, 1% BSA, 0.25 nmol Phalloidin Atto 488 (Sigma–Aldrich) for 25 min at room temperature in the dark. Nuclei were stained with Hoechst 33342 (1 μg/ml) (Sigma–Aldrich). After this, cells were washed six times with PBS and samples were air dried and mounting with BioMount (Bio-Optical). The images were obtained using a confocal microscope TCS SP5 (Leica).

### Purification of actin and estimation of protein concentrations

Actin was isolated as described in [31] from acetone powder prepared according to [32]. Protein concentrations were estimated using molar absorption coefficients (ε^1 mg/ml^_280_) of 1.08 for actin [33] and 1.37 for titin [34].

### Statistical data

The results obtained during the cytotoxicity experiments were statistically evaluated using the Mann–Whitney *U* test with the confidence levels *P* ≤  0.05. Data are given as the mean ± S.E.M.

## RESULTS

### SDS/PAGE and Western blotting of smooth muscle titin

**Figure 1:**
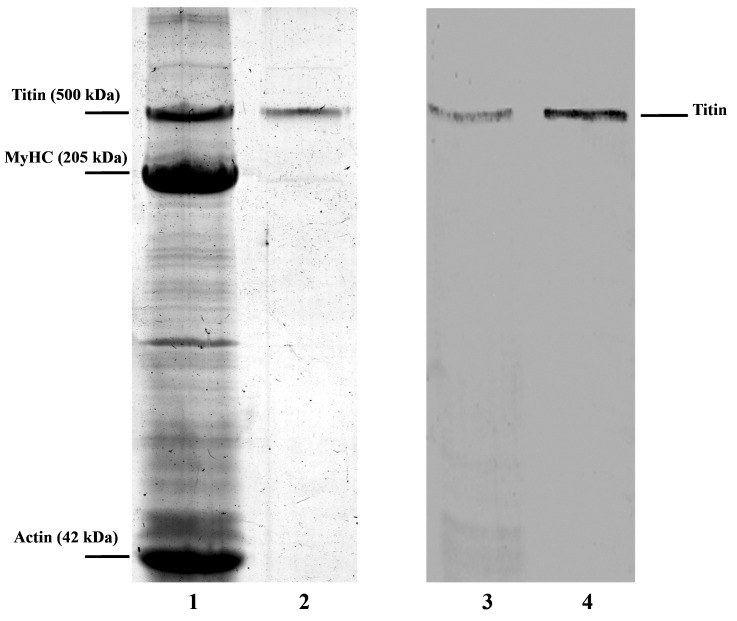
SDS/PAGE and Western blotting of SMT (**1**) Chicken gizzard protein extract. (**2**) SMT preparation purified on a Sepharose-CL2B column. Protein bands for titin, myosin heavy chains (MyHC) and actin are labelled. (**3**) Western blotting of titin with antibodies 9D10 (protein extract). (**4**) Western blotting of purified titin.

Figure 1 shows SDS/PAGE and Western blotting of crude chicken gizzard SMT and the purified preparation. Western blotting with 9D10 antibodies support the identification of this protein as titin (Figure 1, bands 3 and 4) with a molecular mass of approximately 490–500 kDa.

### EM of smooth muscle titin aggregates

**Figure 2:**
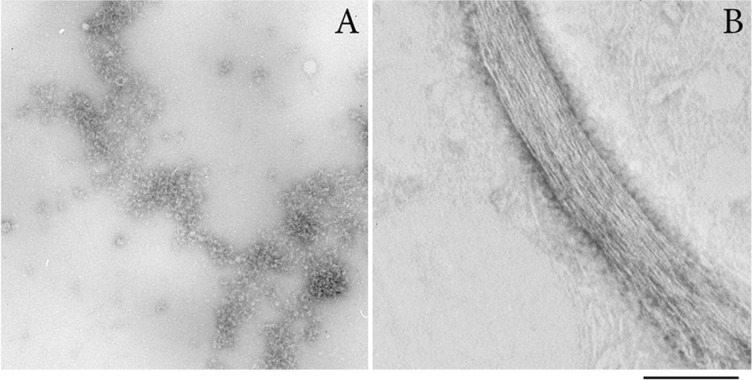
EM of negatively stained SMT aggregates (**A**) Amorphous titin aggregates are the main form of the aggregated protein. (**B**) A bundle of linear fibrils. The width of one fibril in the bundle is approximately 2 nm. SMT aggregates were obtained by 24 h dialysis in 0.15 M glycine–KOH, pH 7.0, at 4°C. Staining used 2% aqueous uranyl acetate, scale 100 nm.

Figure 2 shows EM of negatively stained SMT aggregates. After 24 h incubation at 4°C in 0.15 M glycine–KOH, pH 7.0–7.5, SMT formed amorphous aggregates (Figure 2A) together with compact bundles of linear fibrils. These bundles extend up to several micrometres in length and 100 nm in width (Figure 2B). The fibril bundles were rare and the electron microscope field of view contained mostly amorphous aggregates, the formation of which was also observed in 0.2 M KCl, 10 mM imidazole, pH 7.5 (results not shown).

### Association of smooth muscle titin aggregates with Congo red and thioflavin T

**Figure 3:**
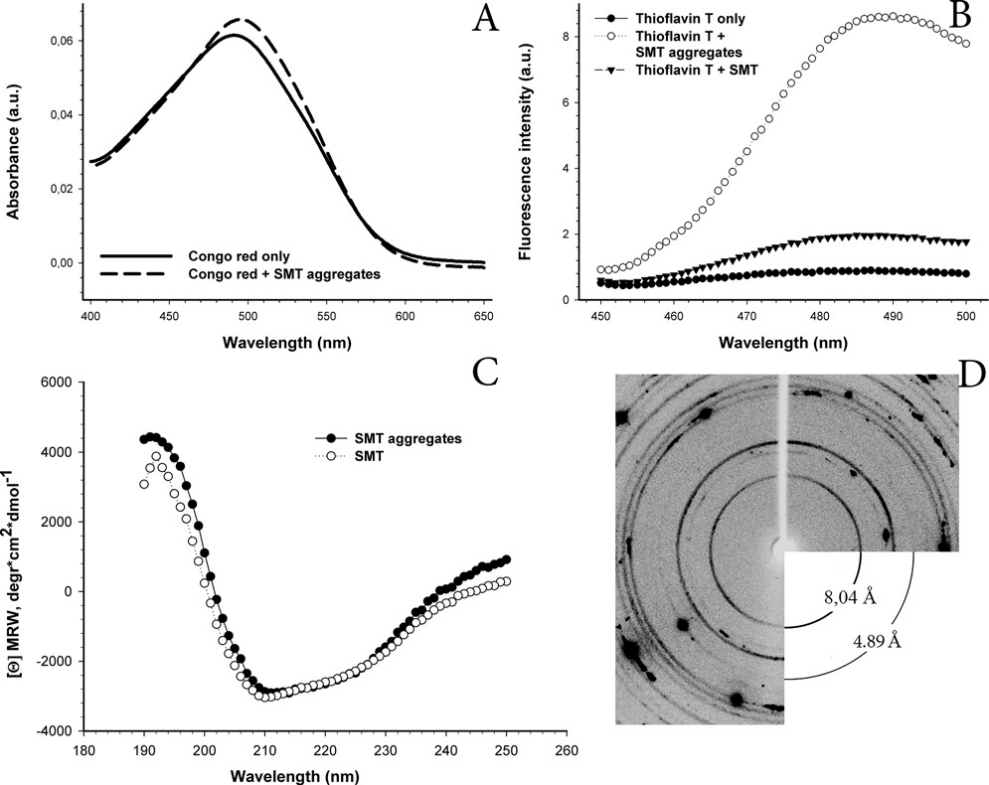
Corroboration of the amyloid nature of SMT aggregates by different methods (**A**) Binding of SMT aggregates to CR. The absorption spectrum of the CR stain is shifted to slightly longer wavelength (dashed line) in the presence of SMT aggregates. SMT aggregates were formed in 0.15 M glycine–KOH, pH 7.0 over 24 h at 4°C. Measurements were done in the same solution. (**B**) ThT staining of SMT aggregates. ThT fluorescence increased in the presence of SMT aggregates compared with that with monodispersed SMT and buffer solution alone. SMT aggregates were formed as above and monitored in the same solution. The measurements were conducted in the same solution. (**C**) CD spectrum of SMT. The α-helix content of non-aggregated SMT in 0.6 M KCl, 30 mM KH_2_PO_4_, pH 7.0 was 6.1%. Helix content of aggregated SMT in 0.15 M glycine, pH 7.5 was 5.5%. (**D**) X-ray diffraction of SMT aggregates. Reflections were detected at: 8.04, 6.09, 5.67, 4.89, 4.11, 3.94, 3.74 and 3.48 Å. The 8.04 Å and 4.89 Å reflections can be ascribed to the β-structure. Reflection 4.11 Å belongs to paraffin on whose surface the studied object was located.

To clarify the possible amyloid nature of the SMT aggregates, CR and ThT were used (Figure 3). Addition of CR solution to SMT aggregates resulted in a characteristic shift in the absorption spectrum from approximately 490 nm to 500 nm (Figure 3A) which is intrinsic to all known amyloid proteins. Fluorescence intensity of ThT increased (7-fold) in the presence of SMT aggregates compared with the monodisperse protein (Figure 3B), which also suggests the aggregates are amyloid.

### CD analysis of smooth muscle titin secondary structure

Figure 3(C) shows CD spectra of SMT before and after the formation of aggregates. No changes were detected in secondary structure on formation of aggregates: the preparation after chromatography had 6.1% α-helix and 40.5% β-structure, whereas helix and β-structure content in aggregated SMT was 5.5% and 40.2%, accordingly. Thus, the results of the CD analysis suggest that the presence of disordered secondary structure, as well as a high β-structure content that may be the reason for an insignificant increase in ThT fluorescence in the monodisperse protein (Figure 3B, thioflavin T+SMT). The data may also explain the simplicity of formation of aggregates *in vitro*.

### X-ray diffraction of smooth muscle titin aggregates

X-ray diffraction of aggregated SMT revealed reflections at: 8.04, 6.09, 5.67, 4.89, 4.11, 3.94, 3.74 and 3.48 Å (Figure 3D). The pronounced reflection 8.04 Å and the weaker at 4.89 Å can be ascribed to β-structure. We clarified that reflection 4.11 belongs to paraffin on the surface of which the object is located (Figure 3D). Other reflections were not being identified. The presence of a cross-β structure identified by X-ray diffraction analysis [35–37] confirms that SMT aggregates are amyloids.

### Dynamic light scattering of smooth muscle titin amyloid aggregates

**Figure 4:**
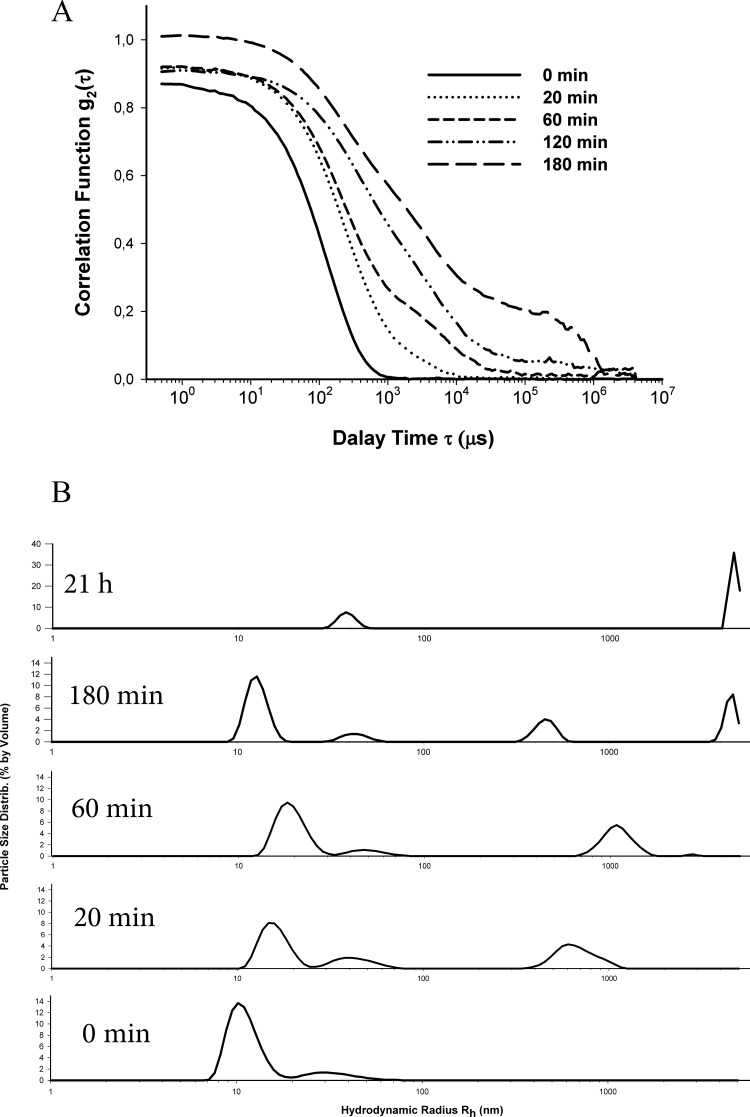
Kinetics of fibril formation by SMT, monitored with DLS (**A**) Evolution of field autocorrelation functions *g*_2_(t) of light scattered during SMT amyloid aggregate formation (pH 7.0–7.5, T=10–25°C). (**B**) Distribution of SMT particles. Generation of large aggregates and their time-dependent growth are shown.

To clarify the time-course of SMT amyloid formation, we used DLS as a less invasive way to detect and characterize the aggregates of different size [38]. Figure 4(A) shows the change in the autocorrelation function of the scattered light upon formation of SMT aggregates at pH 7.0 over 180 min. During the first 60 min incubation, almost mono-exponential decay of the correlation function *g*_2_(t) was observed (Figure 4A), with the subsequent emergence of a shoulder at high correlation times. This indicates the formation of large aggregates with a smaller diffusion coefficient. After 180 min, the correlation function *g*_2_(t) had a more pronounced shoulder (Figure 4A) at high correlation times which indicated the increase in aggregation. Several peaks reflecting the dimensions of the aggregates (Figure 4B) were obtained after the correlation function analysis. Prior to the formation of aggregates, only two well resolved peaks with the average Rh approximately 12–15 nm (the dominating peak of approximately 90%) and 45 nm (the minor peak of approximately 10%) were observed. The first peak corresponds, most likely, to SMT molecules, whereas the second peak may be evidence for an insignificant presence of other proteins in the preparation, for example a high-molecular titin isoform (molecular mass approximately 1.5 MDa), the minor presence of which was recorded by SDS/PAGE (results not shown). The third peak, indicating the formation of titin aggregates (Rh approximately700 nm, Figure 4B) appears after 20 min incubation. The size of this fraction increased during incubation with the eventual emergence (at 180 min) of the fourth peak (Rh approximately 4500 nm, Figure 4B). These fourth peaks indicate the appearance of larger protein aggregates, the number of which increased during the following incubation period. When the experiment terminated (after 21 h), two main fractions with Rh approximately 4500 nm (the main peak corresponding to aggregates) and Rh approximately 40 nm (the minor peak present during the whole experiment) were observed. It should be noted that the peak with Rh approximately 4500 nm was at the limit of the range of this method. Therefore, the formation of larger SMT aggregates cannot be excluded.

### Analysis of the sizes of protofibril nuclei from the concentration dependence of the lag-time and rate of amyloid formations for the I27 immunoglobulin domain from human cardiac titin and actin

To analyse the size of protofibrils nuclei (least stable species on the reaction pathway for formation of fibrils), formed by SMT, the theoretical approach suggested in [39] was used. According to [39], in order to estimate the size of protofibrils nuclei and a possible scenario of formation of titin aggregates, it is necessary to make a number of kinetic experiments, where the only variable parameter is the monomer concentration. Characteristic times *T*_lag_ (the lag-period duration), *T*_2_ (the time of transition of all monomers into fibrils) and *L*_rel_ (the *T*_lag_/*T*_2_ ratio) are calculated for each experimental curve. It was demonstrated that the dependences of log *T*_2_ and *L*_rel_ on log [*M*_Σ_] (the logarithm of the initial concentration of monomers) have a linear character, thereby the values of corresponding slope coefficients for each dependence can be used for computation of the size of folding nuclei of protofibril (including those of non-amyloid type) and for elucidation of the mechanism of aggregate/amyloid formation.

**Figure 5:**
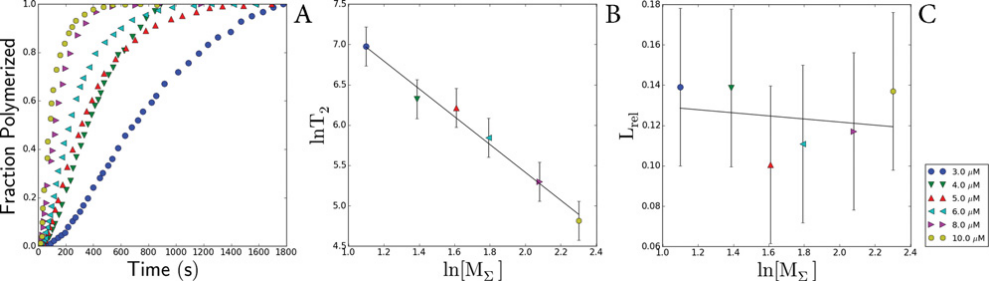
Calculations of the sizes of nuclei for actin fibrils (**A**) Dependence of normalized ThT fluorescence on time for actin [40]. (**B**) Dependence of *L*_rel_ on log *T*_2_. (**C**) Dependence of *L*_rel_ on log [*M*_Σ_]. The slope angle of the fitted line is approximately −0.01, i.e. *L*_rel_ is independent of log [*M*_Σ_]. All *L*_rel_ values are also less than 0.2 which, according to the models of formation of fibrillar aggregates; means that the formation of actin fibrils occurs by the linear mechanism (see [39]). The size of the primary nucleus was calculated from the slope to be *n*=3.4±0.5.

**Figure 6:**
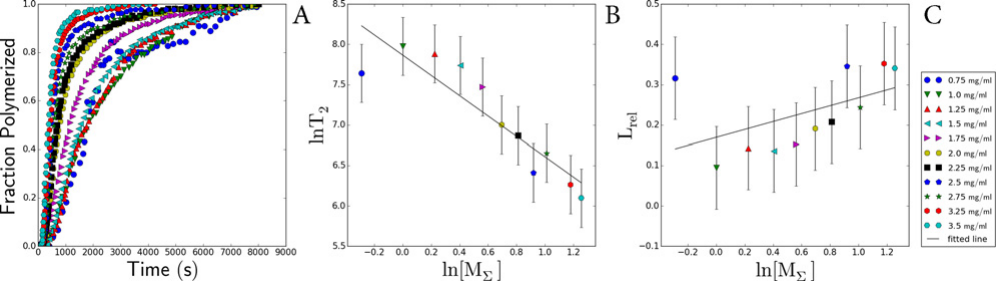
Calculations of the sizes of nuclei for TI27 fibrils (**A**) Dependence of normalized ThT fluorescence on time for TI27 [22]. (**B**) Dependence of log *T*_2_ on log [*M*_Σ_] for TI27. This was used to calculate the size of the secondary nucleus. The slope of the fitted line is approximately −1.26 which, according to formulas from [39], indicates that the size of the secondary nucleus is *n2*=1.5±0.4. (**C**) Dependence of *L*_rel_ on log [*M*_Σ_] for TI27. The slope of the fitted line is −0. 1, i.e. *L*_rel_ is dependent on log [*M*_Σ_]. Some *L*_rel_ values exceed 0.2 and, according to [39], cannot be explained by the linear model. The size of the primary nucleus was calculated based on the size of the secondary nucleus and the slope of the straight line is 2.4±0.5.

In our study, estimations of nuclei sizes and mechanisms of fibrils formation for titin and actin were extrapolated from analysis of the known data of aggregation kinetics for the I27 Ig domain from human cardiac titin (TI27) [22] and kinetic data on the assembly of actin filaments [40]. Both sets of data were digitized, and in the case of TI27, part of the curve was excluded from the analysis. It was found that the growth of amyloids formed by TI27 is exponential, i.e. in addition to the formation of the primary folding nuclei the generation of secondary folding nuclei by the branching mechanism also takes place, accelerating the formation of new fibrils. Formation of actin filaments occurs by linear growth – fibrils increase only due to attachment of monomers to the aggregate ends. This is supported by literature data [40] and by a direct analysis of the kinetic data (Figure 5). In the case of TI27, the conclusion was made based on the analysis of experimental data [22] on amyloid aggregation of protein. According to the theory advanced in [39] for linear growth (observed in actin), the characteristic time of the relative lag-period *L*_rel_ does not exceed 0.2 and is independent of log [*M*_Σ_] as occurs in the case of actin (Figure 5, *L*_rel_ compared with log [*M*_Σ_] for actin) [40]. It is seen from Figure 6 that though the values of *L*_rel_ for TI27 do not exceed 0.2, *L*_rel_ depends on log [*M*_Σ_] that, as mentioned above, cannot occur by a linear mechanism. This effect can be explained by the presence of the secondary nucleus formation *n*_2_ with the size of 1.5±0.4 (Figure 6). It is interesting that in spite of the difference in aggregation mechanism, the size of the primary nucleation core for TI27 is 2.4±0.5 (Figure 6) that approximately (within error) corresponds to the size of the primary nucleus is equal to 3.4±0.5 (Figure 5), calculated for actin [40].

### Cytotoxicity of smooth muscle titin amyloid aggregates

**Figure 7:**
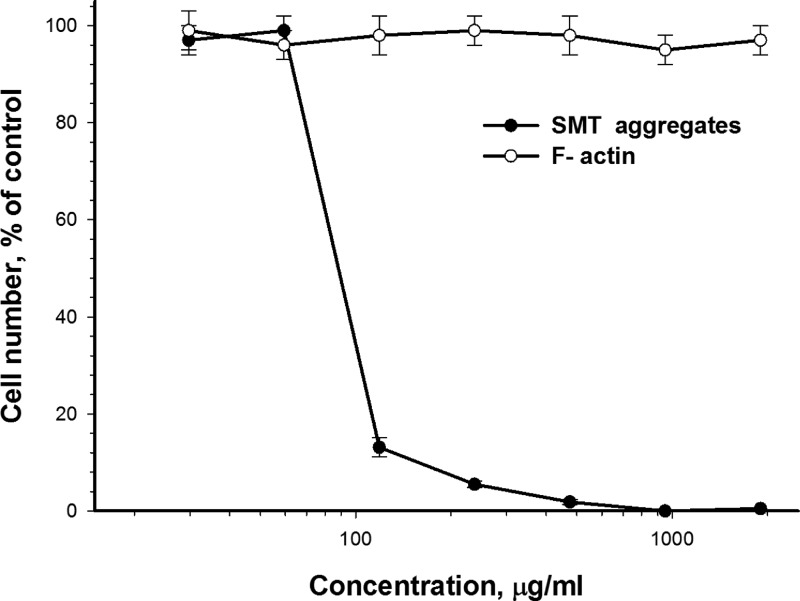
Toxic effect of SMT amyloid aggregates on bovine aortic smooth muscle cells Seventy-two hours incubation, F-actin was used as a control. Values are given as mean ± S.E.M., *n*=5.

**Figure 8:**
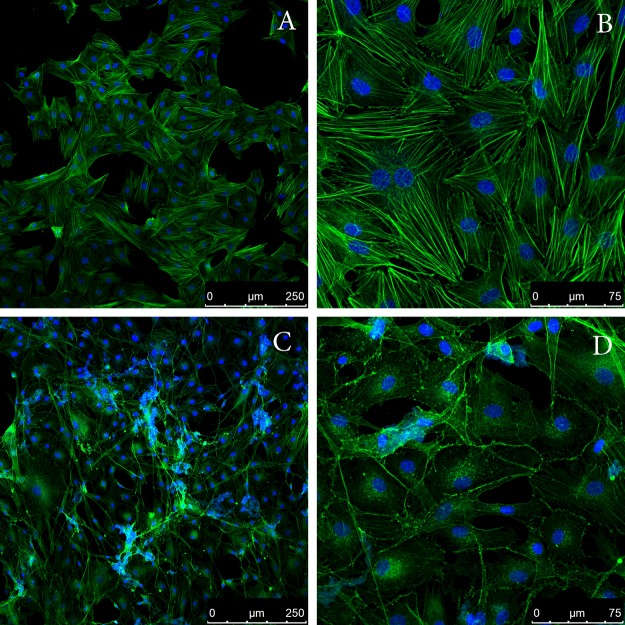
Addition of SMT amyloid to the culture media leads to disorganization of the actin cytoskeleton of aortic smooth muscle cells Panels show confocal microscopy of bovine aortic smooth muscle cells stained with phalloidin Atto 488. (**A** and **B**) Smooth muscle cells in the presence of actin fibrils (control); (**C** and **D**) smooth muscle cells in the presence of SMT amyloid aggregates. Incubation time was 48 h.

Aβ peptides are involved in pathogenesis of Alzheimer's disease. Data are available on the toxic effect of the Aβ(1–40) peptide on smooth muscle cells [41], and this effect is proposed to be associated with the disturbance of adhesion of smooth muscle. SMT is one of the main proteins in the smooth muscle and, as shown by the data here, can relatively quickly form amyloid. Therefore, we studied toxicity of SMT amyloid in smooth muscle cells. Figure 8 shows the cytotoxic action of SMT aggregates on bovine aortic smooth muscle cells. At a SMT concentration approximately 70 μg/ml, the death of 50% cells (IC_50_) was observed (Figure 7). This cytotoxic effect took place only after 72 h incubation of cells together with SMT amyloid aggregates. Prior to these changes in cell adhesion and cell sprawling were observed. For a more detailed investigation of this phenomenon, we studied the changes in the actin cytoskeleton of bovine aortic smooth muscle cells after incubation with SMT amyloid (Figure 8). Confocal microscopy showed disorganization of the actin cytoskeleton upon the addition of SMT amyloid aggregates and until marked toxic effect (Figures 8C and 8D).

## DISCUSSION

Our results demonstrate that titin isolated from smooth muscles of chicken gizzard (Figure 1) can form *in vitro* amorphous aggregates and bundles of linear fibrils in solution containing 0.15 M glycine–KOH, pH 7.0–7.5 (Figure 2). To clarify the amyloid nature of SMT aggregates, we studied their ability to bind to stains for diagnostic for amyloid, CR and ThT, (Figures 3A and 3B) and performed experiments on X-ray diffraction (Figure 3D). As known, interaction of ThT with amyloid leads to an increase in the stain fluorescence [42]. CR is a sulfonic nitro dye with a hydrophobic central part consisting of a bisphenol group located between negatively-charged ends of the dye molecule [43]. Binding of CR to amyloid leads to displacement of the absorption spectrum from approximately 490 nm to 500 nm and our spectra (Figure 3A) showed a similar shift. Corroboration of the presence of a cross-β structure in SMT aggregates was demonstrated using X-ray diffraction, which showed a pronounced 8.04 Å reflection and 4.89 Å reflection (Figure 3D), which indicate cross-β structure [35–37].

Examination of the dynamics of amyloid formation by SMT using DLS revealed fast aggregation (Figure 4). During the initial 20 min phase aggregates with Rh approximately 700 nm formed (Figure 4B). During the following 2.5 h, further aggregation was observed including large aggregates with Rh approximately 4500 nm (Figure 4B). After 21 h, this species become the most abundant but it should be noted that they are at the limit of the measuring range of the DLS method, therefore formation of even larger SMT aggregates cannot be excluded, particularly, bundles of linear fibrils of several micrometres in length.

An analysis of the size of folding nuclei of protofibrils performed with the use of the aggregation kinetic data for the I27 Ig domain from human cardiac titin (TI27) [22] showed that the size of the primary nucleus for TI27 is 2.4±0.5 (Figure 6). This is one monomer larger than the size of the secondary nucleus (1.5±0.4, Figure 6). These data indicate that titin aggregation, including that of SMT, can proceed not by a linear mechanism, as occurs with non-amyloid actin fibrils [40], but by another mechanism involving branching and formation of secondary nuclei on the surface of fibrils. It was shown [22] that Ig titin domains with closely similar sequences (more than 42%) have a high tendency to co-aggregate. These results are not in conflict with our data demonstrating a high rate of SMT aggregation.

Thus, the results of our experiments showed that SMT forms *in vitro* amyloid aggregates over relatively short time intervals. These data together with the high content of the β-sheet structure in SMT (Figure 3C), suggest that SMT could be involved in amyloidosis in smooth muscles. This suggestion is consistent with amyloidosis with other smooth muscle proteins. Amyloid aggregates of SAA (an amyloid-related protein from human serum) and its fragments can be accumulated in the intima and medial arterioles beneath the venular endothelium [7]. Medin amyloids (a 50-aa-long peptide of lactadherin) were discovered in the case of aortic amyloidosis [8]. Data are also available indicating amyloid aggregates of Aβ peptide [44], a considerable cytotoxic effect of which was found in smooth muscle cells [41] that can also contribute to development of aortic amyloidosis. Therefore, our results on the cytotoxic effect of SMT amyloid (Figure 7) support the possibility of involvement of SMT in amyloidogenesis. Cytotoxicity of SMT aggregates may result from their action on the smooth muscles cytoskeleton (Figures 8C and 8D), which plays a vital role in cells development [45]. On the other hand, the cytotoxicity of SMT amyloid aggregates could be mediated by their effect on intercellular contacts, which leads to the change in adhesion and sprawling of cells observed in our experiments.

Summarizing our results, we can say the following: Our *in vitro* studies indicate that under conditions close to physiological and at neutral pH, SMT (500 kDa) generates amyloid aggregates. These aggregates have a pronounced cytotoxic effect on smooth muscle cells accompanied by distortions of the actin cytoskeleton and cell adhesion. The *in vitro* aggregates form over short time intervals (tens of minutes) thus, their occurrence in human and animal organs and cells cannot be excluded during development of pathologies, in particular, amyloidosis. Further research is needed to establish the possible role of SMT amyloid depositions in development of amyloidosis muscle tissue.

## Abbreviations

Aβ: amyloid-β
CR: Congo red
DLS: dynamic light scattering
FnIII: fibronectin III-like domains of titin
Ig: immunoglobulin-like domains of titin
Ig: immunoglobulin
Rh: hydrodynamic radius
SAA: serum amyloid A
SMT: smooth muscle titin
ThT: thioflavin T
